# Identification of pan-kinase-family inhibitors using graph convolutional networks to reveal family-sensitive pre-moieties

**DOI:** 10.1186/s12859-022-04773-0

**Published:** 2022-06-22

**Authors:** Xiang-Yu Lin, Yu-Wei Huang, You-Wei Fan, Yun-Ti Chen, Nikhil Pathak, Yen-Chao Hsu, Jinn-Moon Yang

**Affiliations:** 1 Institute of Bioinformatics and Systems Biology, National Yang Ming Chiao Tung University, Hsinchu, Taiwan; 2 Institute of Biomedical Engineering, National Yang Ming Chiao Tung University, Hsinchu, Taiwan; 3 Institute of Molecular Medicine and Bioengineering, National Yang Ming Chiao Tung University, Hsinchu, Taiwan; 4 Institute of Bioinformatics and Structural Biology, National Tsing Hua University, Hsinchu, Taiwan

**Keywords:** Pan-kinase family inhibitor, Graph convolutional network, Visualized explanation, Gradient-weighted class activation mapping, Family-sensitive pre-moiety

## Abstract

**Background:**

Human protein kinases, the key players in phosphoryl signal transduction, have been actively investigated as drug targets for complex diseases such as cancer, immune disorders, and Alzheimer’s disease, with more than 60 successful drugs developed in the past 30 years. However, many of these single-kinase inhibitors show low efficacy and drug resistance has become an issue. Owing to the occurrence of highly conserved catalytic sites and shared signaling pathways within a kinase family, multi-target kinase inhibitors have attracted attention.

**Results:**

To design and identify such pan-kinase family inhibitors (PKFIs), we proposed PKFI sets for eight families using 200,000 experimental bioactivity data points and applied a graph convolutional network (GCN) to build classification models. Furthermore, we identified and extracted family-sensitive (only present in a family) pre-moieties (parts of complete moieties) by utilizing a visualized explanation (i.e., where the model focuses on each input) method for deep learning, gradient-weighted class activation mapping (Grad-CAM).

**Conclusions:**

This study is the first to propose the PKFI sets, and our results point out and validate the power of GCN models in understanding the pre-moieties of PKFIs within and across different kinase families. Moreover, we highlight the discoverability of family-sensitive pre-moieties in PKFI identification and drug design.

## Introduction

Over 300 protein kinases share a common biological function as ATP-dependent phosphorylation enzymes [1], with a significant role in signal transduction, particularly in the progression of complex diseases such as cancers [2], immune system misfunctions, and Alzheimer’s disease [3]. Accordingly, they fall under the category of intensively investigated drug targets [2, 4], with 61 US Food and Drug Administration (FDA)-approved kinase inhibitors to date [5]. Due to the highly conserved catalytic sites of protein kinases, investigation of kinase inhibitor selectivity in the kinome space has been a challenge [6]. On the contrary, protein kinases within a single kinase family regulate shared cancer-related pathways [7]; therefore, inhibition of a single target leads to drug adaptation and resistance [8–12].

To overcome these issues of drug resistance, various studies have suggested drug combinations or multi-targeting drugs to be an effective approach for complex diseases [8, 9, 13–18]. Moreover, several approved kinase inhibitors were originally designed as pan-kinase-family inhibitors (PKFIs) to target multiple proteins of the kinase families [16, 17, 19–21], such as the epidermal growth factor receptor (EGFR)/HER2 dual-targeting inhibitor lapatinib [19] and the pan-vascular endothelial growth factor (VEGF) inhibitor sorafenib [22, 23]. To discover potential inhibitors within the large chemical space, deep learning techniques have been applied to rapidly identify potential inhibitors against a single target within the kinome [21, 24, 25]; however, these studies have seldom addressed the multi-targeting issue or the lack of explainability for the trained model’s judgment.

Graph convolutional network (GCN)[26] is a recently developed deep learning architecture that is designed to extract the spectrum information on the topological data. Due to the no-distanced and no-ordered properties of the topological data, it is hard to be operated by previous machine learning and deep learning techniques until the GCN architecture is brought onto the stage. The power that the GCN model provides is on the capability of self-organizing the surrounding information of each atom in the compound, and extracting the chemical substructures with different sizes. Therefore, with the help of GCN architecture, now we have the chance to achieve our aim: self-organizing the pre-moieties within families without using pre-defined fingerprints. On the other hand, some explainability methods, like gradient-weighted CAM (Grad-CAM)[27], for GCNs were developed to help identify functional groups or substructures on small molecules for biological molecular properties.

In this study, we aimed to develop GCN models to identify PKFIs and to highlight the chemical pre-moieties. First, we collected PKFIs from the ChEMBL database [28, 29] and kinase profiling data [30], and a total of 60,122 compounds of 384 kinases from 103 families within 195,802 data points were obtained. We then selected two families for each of the four kinase groups, tyrosine kinase (TK), AGC, CMGC, and calmodulin-dependent protein kinase (CAMK), and built GCN models for each selected family. Then, we applied gradient-weighted class activation mapping (Grad-CAM) [27] method to explain each inhibitor’s prediction. Our results indicate that our GCN model can aid in judging the viability of identifying family-sensitive pre-moieties in PKFIs.

**Fig. 1:**
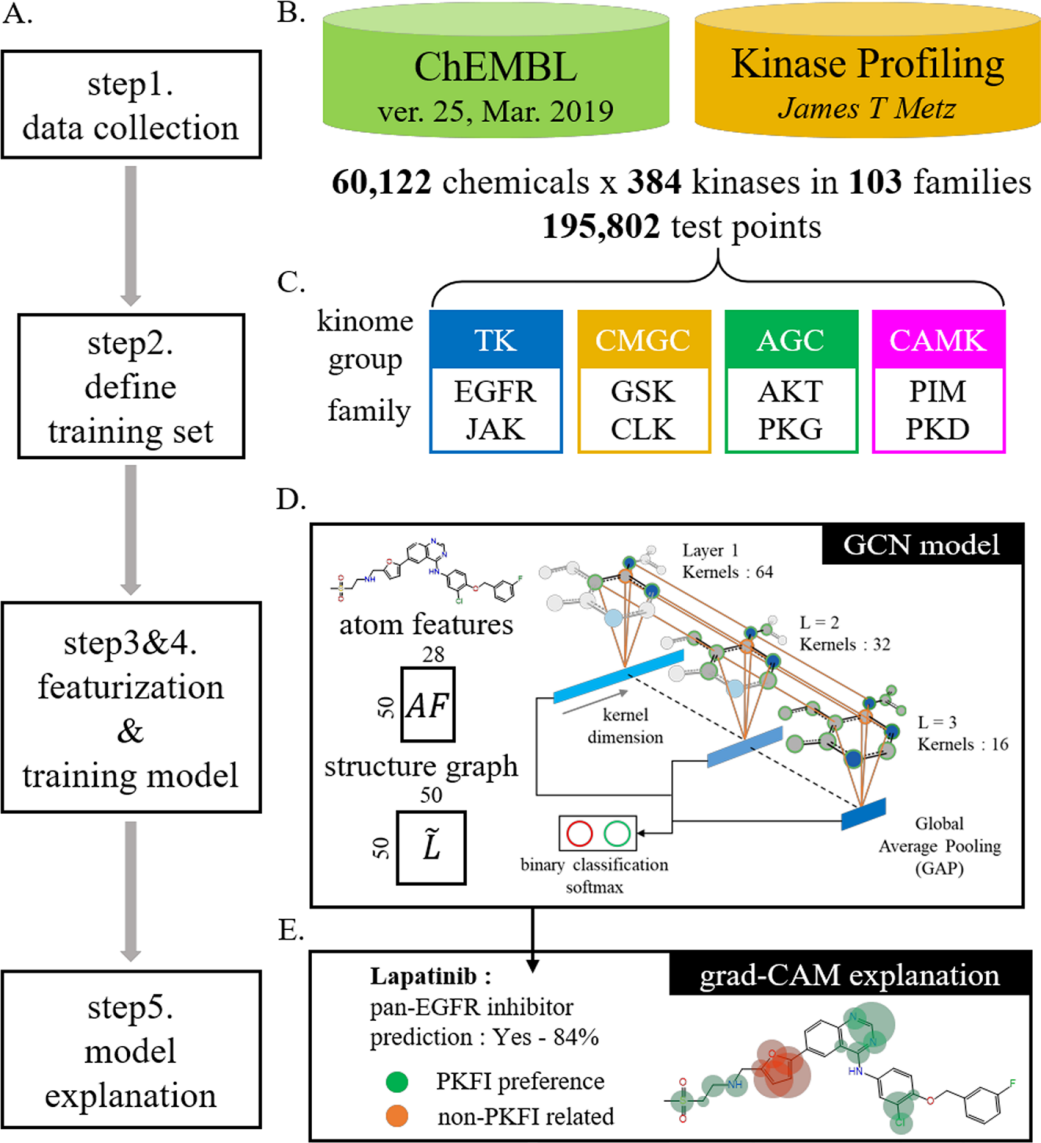
Scheme for utilizing atom-wise featurization and topological information on compounds for the identification of pan-kinase family inhibitors (PKFIs) using graph convolutional network (GCN) models. **A** Schematic of the research framework. **B** 195,802 test datasets of 60,122 chemicals and 384 kinases were collected from the ChEMBL database and kinase profiling. **C** Eight families in four kinase groups were targeted in this study. **D** Each compound is transformed into atom features and a structure graph for GCN architectures to identify PKFIs. **E** A visualized explanation was made using the grad-CAM method

An overview of our method and models for identifying PKFIs is presented in Fig. 1. First, we collected 195,802 sets of kinase-compound activity data and defined the PKFI sets, followed by the featurization of each inhibitor into atomic features and Laplacian matrix-based topologies. The GCN model was constructed for each family to identify the PKFIs. Three indices, accuracy, the area under the receiver operating characteristic curve (AUROC), and Matthews correlation coefficient (MCC), were used in addition to applying the Grad-CAM sample-wise explanation to examine the predictability and reliability of our models and to further determine the family-sensitive pre-moieties.

## Results

### PKFI sets and model performance

To assess the PKFI differences between each kinase family, we applied GCN models on eight families in four kinase groups (Table 1) individually. The configuration of our GCN architecture includes three graph convolutional layers with 64, 32, and 16 kernels respectively, and all. All convolutional feature maps were followed by a GAP layer, and then concatenate all GAP feature vectors were concatenated before applying a softmax classifier (Fig. 5). Each model was trained for 100 epochs, with an 80:20 training/testing distribution of each PKFI set utilizing the ADAM optimizer with a learning rate of 0.001, β_1 = “0.9”, β_2 = “0.999” based on cross-entropy as a loss function. All models were implemented in Keras [35] with a Tensorflow backend [36].

**Table 1:** Performance on eight PKFI sets

Group	Family	Members	Data size		ACC	MCC	AUROC
			Positive	Negative			
TK	EGFR	4	620	808	0.85	0.70	0.92
	JAK	4	1347	877	0.84	0.64	0.91
CAMK	PIM	3	688	628	0.84	0.68	0.91
	PKD	3	67	462	0.92	0.16	0.91
AGC	AKT	3	394	772	0.90	0.79	0.94
	PKG	2	51	396	0.89	0.31	0.91
CMGC	GSK	2	243	657	0.75	0.28	0.81
	CLK	4	178	418	0.72	0.20	0.79

To evaluate the performance of each model, three metrics were applied: (1) accuracy (ACC) for general precision, (2) Matthew correlation coefficient (MCC) for measuring the quality of classification according to class-wise distributions, and (3) AUROC for measuring the composite index of sensitivity and specificity. The evaluation metrics of the test results of the eight PKFI sets are presented in Table 1. The average ACC (0.84) and AUROC (0.89) of all models were high enough to distinguish PKFIs. However, the MCC scores of the four GCN models were significantly lower in terms of their misbalanced distribution in the training sets: protein kinase D (PKD) family in the CAMK group (balance: 0.15 positive/negative), cGMP-dependent protein kinase (PKG) family in the AGC group (balance: 0.13), GSK family (balance: 0.37), and CLK family (balance: 0.42) in the CMGC group. Overall, the average ratio of balance of eight families was 0.62, where the balance values of the other four families were all above 0.5 and showed qualified MCC scores. This indicates that increasing the data size may aid in overcoming the imbalanced data distribution.

### Discover common/specific pre-moieties across kinase families

**Fig. 2:**
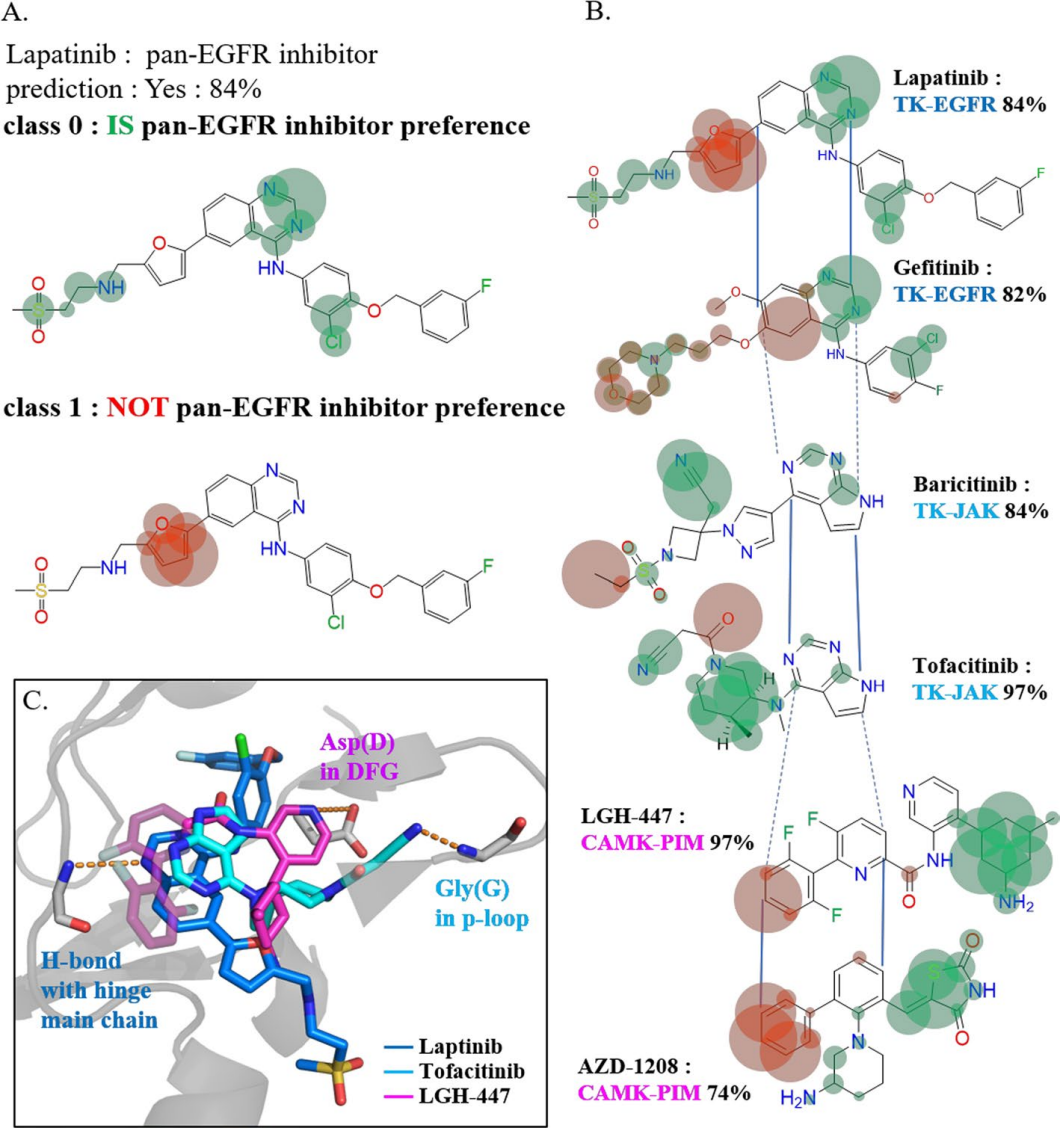
Explanation of the GCN model’s prediction of lapatinib and other inhibitors in EGFR, JAK, and PIM models. (**A**) Grad-CAM preferences of lapatinib from the latest graph convolutional layer for both positive and negative classes. Circles are centered at each atom, with green ones for the positive class and orange for the negative class. The larger the circle, the more the atom contributes to the prediction of the model at a specific class. (**B**) Preferences for different inhibitors within and across families. Within the same family, conserved attention on similar environments is visualized, and family-sensitive pre-moieties can be seen by comparing cross-family inhibitors. (**C**) Crystallized complexes of the pan-EGFR inhibitor lapatinib (deep blue, PDB ID: 1XKK), pan-JAK inhibitor tofacitinib (light blue, PDB ID: 3EYG), and pan-PIM inhibitor LGH-447 (purple, PDB ID: 5DWR) demonstrated three different modes of kinase inhibition

Explanation and visualization were generated using the Grad-CAM method (see method, Eq. (6) and (7)). As shown in Fig. 2, we compared different explanations based on three families: the EGFR family [37, 38], the Janus kinase (JAK) family [39, 40] in the TK group, and the serine/threonine kinase PIM family [41] in the CAMK group. To demonstrate the rationality of the Grad-CAM explanation, lapatinib, a highly selective [42] EGFR/HER2 targeting dual inhibitor used in the treatment of breast cancer [43, 44], was introduced and explained by the EGFR model (Fig. 2). The preferences of the positive class are highlighted by green circles (Fig. 2A). The preference on the double nitrogen atoms in ‘middle naphthalenyl structure’ (structure of aromatic double ring) indicates that the hydrogen bond formable environments on aromatic rings were preserved in pan-EGFR inhibitors and could be captured by the EGFR model. We refer to these conserved chemical environments as family-sensitive pre-moieties, as they only retain parts from fixed moieties; the chemical characteristics are already indicated. This observation has also been validated in X-ray-crystalized complexes wherein the naphthalenyl nitrogen actually interacts with the main chain atoms of the hinge region allowing hydrogen bonding, thereby enabling ATP-competitive interaction to block the kinase activity, which is one of the key modes for designing kinase inhibitors [2, 45] (see Fig. 2C, deep blue).

After confirming the rationality of the explanations of Grad-CAM, we further compared the rationality of the explanations of inhibitors from different families (see Fig. 2B). The EGFR model pays intense attention to the hinge-interacting region, which is also represented in JAK family inhibitors. Explanations of baricitinib [46] and tofacitinib [47] indicate that this region has a minor effect on the formation of pan-family selectivity of JAKs, but is critical for selectivity of the EGFR family. In contrast, the major highlight of JAK inhibitors appears as triple-bonded nitrogen, which interacts with the p-loop structure (see Fig. 2C, light blue). This structure is essential for the transfer of phosphate onto substrates and disrupting it will interfere with phosphorylation. Indeed, within the same kinase group, inhibitors of EGFR and JAK families share partially similar structures but use different modes to interrupt kinase activity, as indicated by the explanation of our models as well as crystallized 3D structures.

In addition to the intra-TK-grouped comparison, we examined the inhibitors of the PIM family in the CAMK group to further assess the inter-grouped differentiation on the explanation of PKFIs. Aligned by the position of the hinge region as the center, LGH-447 and AZD-1208[21] of the PIM family showed no preference for the hinge region, but instead showed a preference for the cyclo-nitrogen regions of the tail (see Fig. 2B). These highlighted pre-moieties in PIM inhibitors (Fig. 2C, purple compound) actually undergo the third mode of interaction with the DFG motif of a kinase to destabilize the kinase structure and further interfere with its function [48].

Through the comparison of inhibitors across three families, family-sensitive pre-moieties and environments were demonstrated, along with the actual kinase-binding inhibitor structures.

### Correlation of model explanation and statistics through current moiety-based fingerprint mapping

**Fig. 3:**
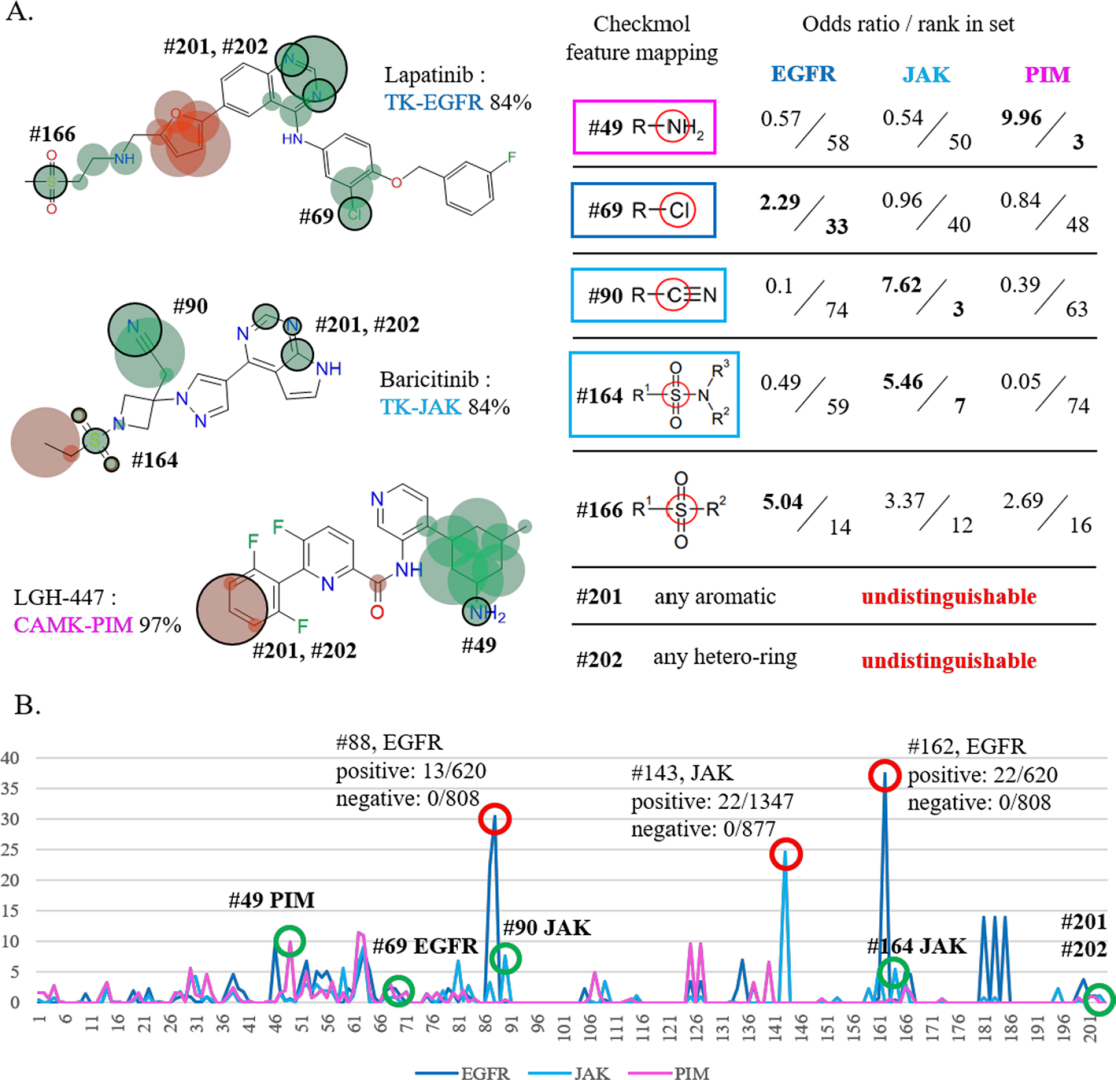
Correlation between preferences and the mapping of current checkmol fingerprints. (**A**) The mapped region of checkmol fingerprint and their odds-ratio ranking (epsilon = 0.5, is added to prevent dividing by zero) of 204 checkmol descriptors within each family of the PKFIs set. Most pre-moieties associated with different modes of kinase inhibition (described previously) are mappable and correlated with the feature distribution in the training sets. However, several pre-moieties are still not precisely defined in the current fingerprint and thus are undistinguishable (i.e., naphthalenyl-nitrogen regions on lapatinib). (**B**) Overall odds-ratio distribution of checkmol descriptors on EGFR, JAK, and PIM datasets with mapped moieties is indicated. It should be noted that several super peaks are observed with relatively few compounds and thus are not currently discussed

While revealing the family-sensitive pre-moieties (defined in Results, section B) by focusing along the decision process of the model, statistical significance must also be considered. Owing to the unfixed structure of pre-moieties, we utilized moiety-based fingerprint checkmol [49] to establish the statistical significance using odds ratio, as shown in Fig. 3. Most discovered pre-moieties are distinguishable (outline fingerprint in Fig. 3A) by checkmol descriptors, such as the triple-bonded nitrogen on baricitinib mapped to the #90 fingerprint and the cyclo-nitrogen on LGH-447 to #49 fingerprint, and also are significantly possessed by positive inhibitors within each PKFI set (i.e., #49 fingerprint concentrative possessed by pan-PIM inhibitors and #90 by pan-JAK inhibitors) (see Fig. 3A). However, there still remain several unmappable pre-moieties. For the naphthalenyl-nitrogen region of lapatinib, the only matched checkmol descriptor is #201 (any aromatic atoms) and #202 (any hetero-ring structures), and both are undistinguishable not only within the EGFR set but also across different PKFI sets (Fig. 3B).

The inconsistency between Grad-CAM-based family-sensitive pre-moieties and moiety-based checkmol fingerprint mapping might indicate that the information and rules of kinase-inhibitor interactions are hidden within the compound structures and compositions to a greater extent than we assumed. The GCN architecture is not only able to discover the specified local environments within inhibitors from each family but also shows the limitations of the current moiety-based describing methods. Furthermore, in this study, the GCN architecture has the potential to broaden the recognizable chemical moiety spaces and thus facilitate the rapid identification of potential inhibitors from large chemical spaces.

## Conclusion and discussion

In this study for exploring kinase multi-targeting, we formulated pan-kinase-family inhibitor sets for the first time and used GCN architecture to identify the hidden information in the PKFI sets of EGFR, JAK, and PIM families. We further applied Grad-CAM to visualize the effects of chemical environments on inhibitors that were considered by the models to make decisions. Validated by the kinase-inhibitor complexes, we discovered that the family-sensitive pre-moieties contain information on kinase-inhibitor interactions and are associated with different modes of inhibition of kinase activity. By comparing our discovered pre-moieties and the checkmol moiety-based fingerprints, we demonstrated the insufficiency of current moiety-based descriptors, which can be overcome by the GCN architecture for recognizing family-specific chemical environments. In summary, the GCN technique has the power to identify PKFIs and learn the undefined pre-moieties in the field of potential drug design and optimization.

## Methods

### Datasets

To access the information on kinase-inhibitor reactivity, we collected 195,802 sets of kinase-chemical activity data from ChEMBL and kinase profiling, which contain about 60,122 compounds belonging to 384 kinases grouped into 103 kinase families.

The ChEMBL data was obtained from the ChEMBL version 25 (March 2019), which contains 15 million data points of 1.8 million chemicals and 12.5 thousand targets. We filtered our kinase-inhibitor set according to the following criteria: (1) selection of targets of 518 kinases [1] by UniProt ID with IC_50_ bioactivity, (2) exclusion of relation of “ ~ ” and “ >  > ” for a certain activity, and (3) use of “Binding” assay type, “SINGLE PROTEIN” target type and confidence score 9 for retaining the direct experimental kinase-inhibitor interaction data. The resulting set contained 95,462 data points for 58,846 compounds and 382 kinases with IC_50_ < 500 nM as the activity cutoff.

To understand the complete test results between compounds and kinases, we collected kinase profiling data (containing 172 kinases and 3,858 compounds) published by Metz [30] and further applied the criteria below to obtain a reliable kinase-inhibitor set: (1) removal of ID-lacking, InChIKey-lacking, and InChIKey-duplicated chemicals, and (2) exclusion of pairs of blank activity results. The filtered dataset contained 1,421 compounds and 172 kinases, with a total of 100,786 test points and pKi > 6 as the activity cutoff.

After collecting these two sources of data, we merged them with “80% voting” for duplicates, which meant that the final active/inactive labels for the duplicated data points were in agreement with 80%-consistent answers among its duplicates, and those with maximum consistency below 80% were excluded. The final kinase bioactivity dataset contained 195,802 test points, with 60,122 compounds and 384 kinases.

### 4.2 Definition of pan-kinase-family inhibitors (PKFIs)

To establish the PKFI sets from kinase-inhibitor data, we further investigated the criteria for any compound $C_{i}$ tested in the kinase family $kF_{i}$ to be a PKFI.

1 $$Test_{{C_{i} \leftrightarrow kF_{j} }} \equiv \left\{ {Test\left( {C_{i} \leftrightarrow {\text{kinases}} \in kF_{j} } \right)} \right\}$$

2 $$Test_{{C_{i} \leftrightarrow kF_{j} }} \to \left\| {Test_{{C_{i} \leftrightarrow kF_{j} }} } \right\| \ge \frac{1}{2}\left\| {kF_{j} } \right\| \ge 2$$

3 $$Label_{{C_{i} }} = \left\{ {\begin{array}{*{20}l} {0,} \hfill & {Test_{{C_{i} \leftrightarrow kF_{j} }} = {\text{active,}}} \hfill \\ {1,} \hfill & {Test_{{C_{i} \leftrightarrow kF_{j} }} = {\text{inactive}}.} \hfill \\ \end{array} } \right.$$

where the total test points within compound *i* and family *j* must be no less than half of the total membership of family *j* with testing data on at least two kinase members. The final label of each PKFI is given as an answer when all the test points are consistently active or inactive.

### Featurization of input compounds

**Fig. 4:**
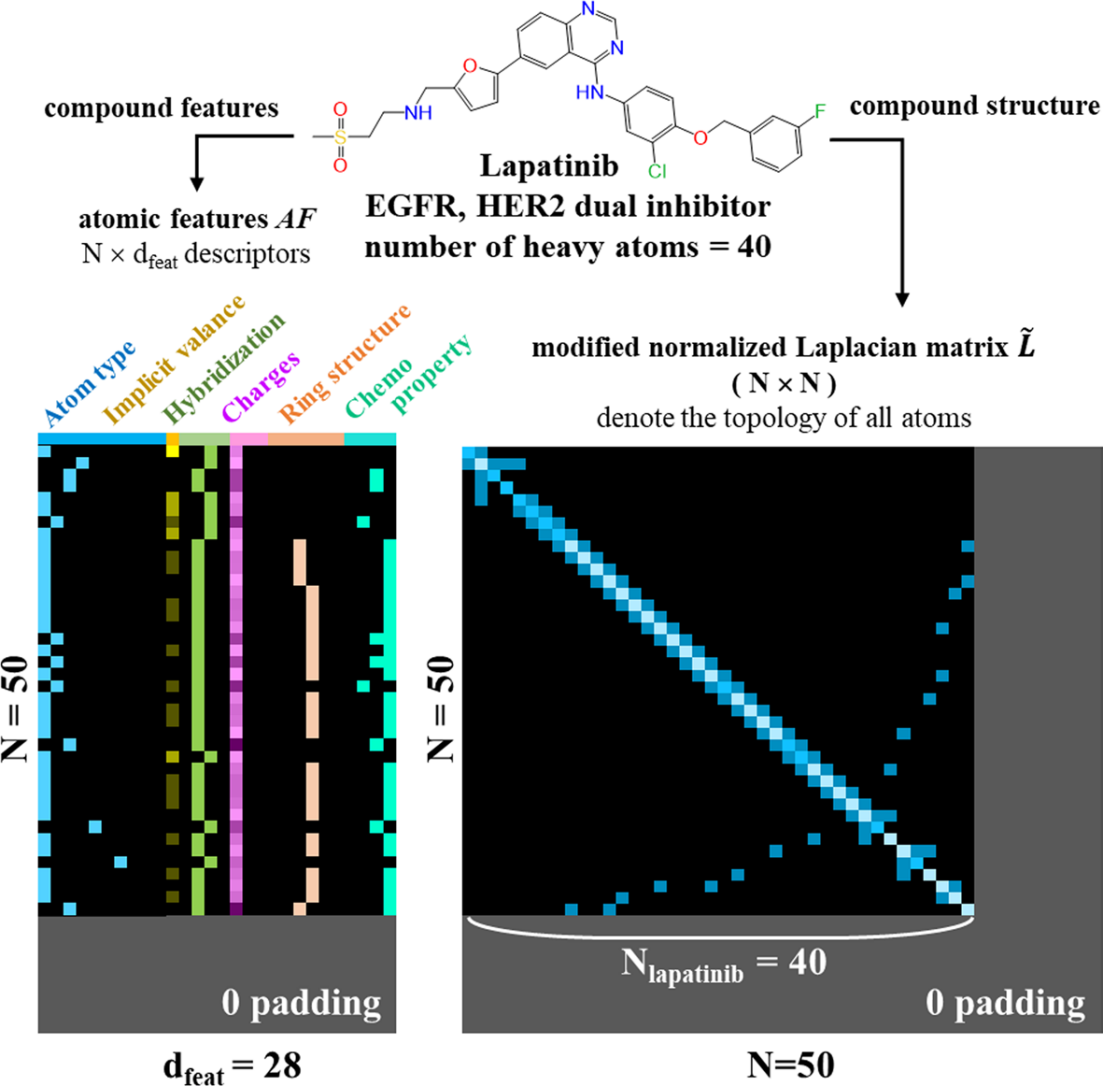
Feature encoding of the input compounds. Each compound is encoded by atomic features and a structure graph. Atomic features contain 28 descriptors belonging to six types, including atom types, hybridization, charges, and chemo-properties. For a structure graph, a modified normalized Laplacian matrix (Eq. (4)) was applied as a representation of the compound’s topology information. Padding to 50 atoms with zeros was applied to contain variable numbers of atoms in the input compounds

To facilitate the classification of PKFIs with GCN frameworks, an attributed graph ${\mathcal{G}}_{i} = \left( {AF_{i} ,\tilde{L}_{i} } \right)$ is presented for each input compound *Ci* where $AF_{i} \in {\mathbb{R}}^{{N \times d_{feat} }}$ is the node descriptions of atomic environments in the compounds, and atom types, chemo-properties, and charges, are described [26, 31] (Table 2). Following the previous work of Kipf and Welling [32], we used a modified normalized Laplacian matrix $\tilde{L}_{i} \in {\mathbb{R}}^{N \times N}$ that encodes the topological structure of connections and bond order cross atoms (Fig. 4):

4 $$\tilde{L}_{i} = \tilde{D}^{{ - \frac{1}{2}}} \tilde{A}\tilde{D}^{{ - \frac{1}{2}}}$$

where $\tilde{A} = A + I_{N}$ is the adjacency matrix of input compounds added by self-interaction, $I_{N} \in {\mathbb{R}}^{N \times N}$ is the identity matrix, and $\tilde{D}_{ii} = \mathop \sum \nolimits_{j} \tilde{A}_{ij}$ is the diagonal degree matrix based on $\tilde{A}$. Given that the task of our GCN models is to identify PKFIs that potentially contain a different number of atoms, we enabled both the atomic environment AF and modified Laplacian matrix $\tilde{L}$ to contain 50 heavy atoms (hydrogen excluded) maximally by padding up the blank region with zeros (Fig. 4).

**Table 2:** Summary of 28 atom descriptions of a compound

Feature	Description	Size
Atom type	C, N, O, S, F, P, Cl, Br, I and other (one-hot)	10
Implicit valance	Bonding hydrogens (integer)	1
Hybridization	sp, sp^2^, sp^3^ and other hybridization (one-hot)	4
Charges	Formal charge (integer)	1
	Partial charge (float)	1
	Radical electrons (integer)	1
Ring structure	The atom is included in rings of size (3–8) (binary)	6
Chemo-property	Chirality: Is the atom a chiral center or not (one-hot)	1
	Aromatic: Is the atom in an aromatic system (one-hot)	1
	Hydrogen bonding: Is the atom a hydrogen bond donor and/or an acceptor (binary)	2
	Total	28

### Graph convolution network architecture

**Fig. 5:**
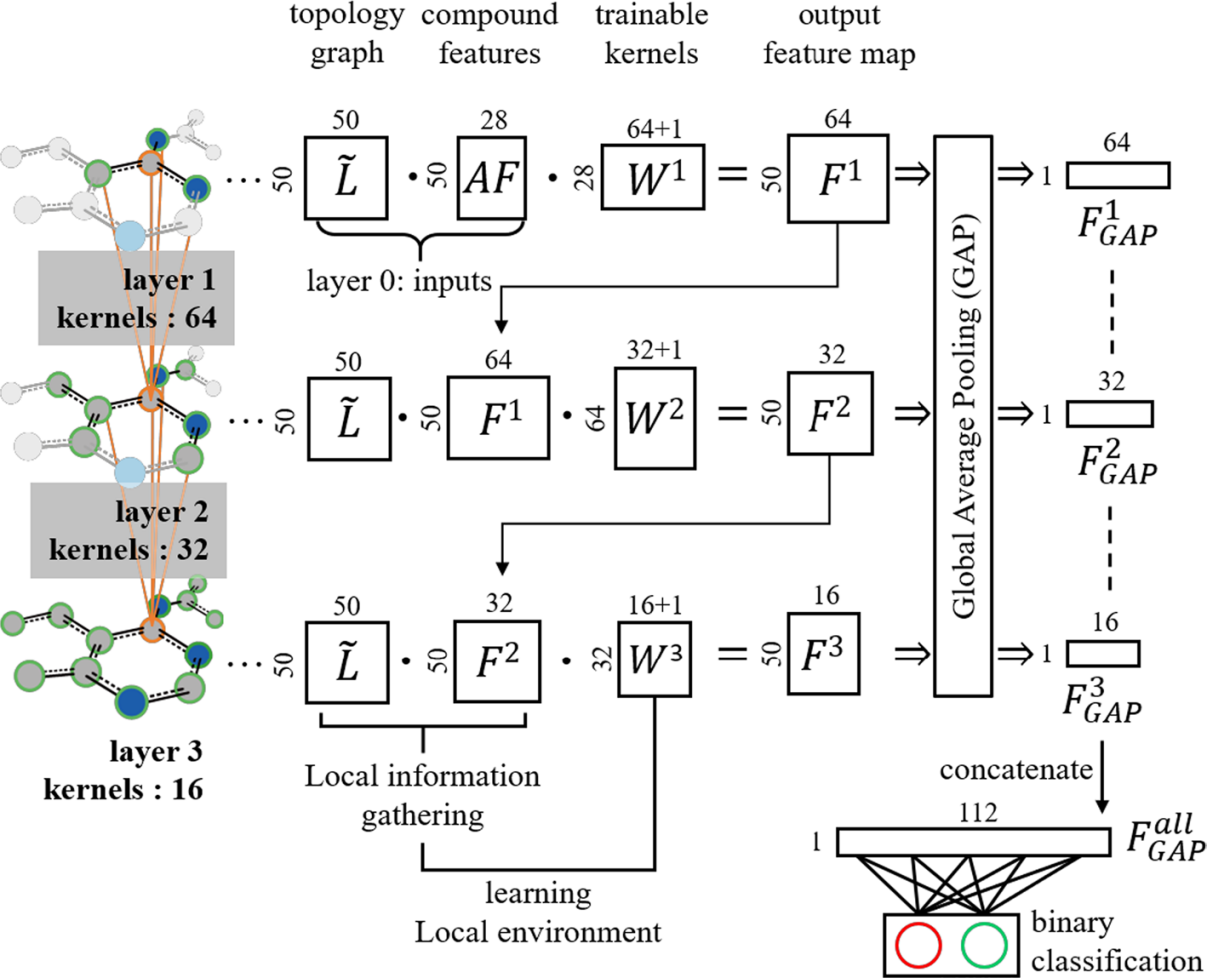
The operation of the graph convolution and GCN model architecture of binary classification is described in this paper. To include the surrounding environments of each atom (local environments), the multiplication of topology graph $\tilde{L}$ and the compound features (either the input atomic features *AF* or the feature map *F*^(*l*−1)^ from the last layer) is required, which should be further multiplied by *W*_*l*_ to learn the information provided by the local environment.

Following the presentation of ${\mathcal{G}} = \left( {AF,\tilde{L}} \right)$ for each input compound, the function of the graph convolution layer is defined as follows:

5 $$F^{l} \left( {AF,\tilde{L}} \right) = \tilde{L} \cdot F^{(l - 1)} \left( {AF,\tilde{L}} \right) \cdot W^{l}$$

where $F^{l}$ denotes the graph convolutional function at layer $l$ and $F^{0} = AF$ is the compound atomic environment, and, $W^{l} \in {\mathbb{R}}^{{d_{l - 1} \times d_{l} }}$ is the trainable kernel set of the *l*th layer that responds to the spectral pattern recognition of compound local information. Figure 5 describes the operation of graph convolution upon the presentation of ${\mathcal{G}} = \left( {AF,\tilde{L}} \right)$ and the schematic of our GCN architecture. To understand the chemical environments of compounds that cannot be provided by atomic features, we utilized the atomic connection information provided by the modified Laplacian matrix $\tilde{L}$ to gather the surrounding information of each atom and form local environments, which is the purpose of equation $\tilde{L} \cdot F^{{\left( {l - 1} \right)}} \left( {AF,\widetilde{ L}} \right)$ (Eq. (5) & Fig. 5). To learn the composition of local environments, trainable kernel weights $W^{l}$ were applied.

As the graph convolution layer is stacked along with the graph, the local environment centered on each atom expands with bond orders. Therefore, to facilitate the sensing of diverse spectral patterns from different sizes of local environments, our GCN model considered all outputs from the three graph convolution layers. The global average pooling (GAP) was then applied after each convolution output to eliminate overfitting upon the pseudo-atomic order stipulated just for utilizing the GCN framework. Then, the final softmax binary classifier was applied to obtain a concatenation of three pooled feature maps to predict PKFIs (Fig. 5).

### Gradient-weighted class activation mapping as model explainability

Grad-CAM was originally designed and applied for convolutional neural networks (CNNs) [33], is an additional explanation for deep learning models that visualize the heat map of the attention region (which contributes the most to compounds for the prediction of the model) from the feature maps of each layer. Owing to the commonality of convolution and graph convolution, Pope and Kolouri further extended it to GCN frameworks [34]. The Grad-CAM method consists of two major steps. We first defined the class-specific weights $\alpha$ for the $k^{th}$ feature of class $c$ at layer $l$:

6 $$\alpha_{k}^{l,c} = \frac{1}{N}\sum\limits_{n = 1}^{N} {{\text{ ReLU }}\left( {\frac{{\partial y^{c} }}{{\partial F_{k,n}^{l} }}} \right)}$$

where $y^{c} \leftarrow GCN\left( {AF_{i} ,{ }\tilde{L}_{i} } \right)$ is the predicted probability on class *c* of compound *i*, and the weight vector ${\upalpha }_{k}^{l,c}$ of *k* features is calculated by the summation of the back-propagated gradients along the atom order dimension.

By defining the weights for each feature *k* of compound *i*, we computed the Grad-CAM feature map of layer $l$ through ${\upalpha }_{k}^{l,c}$:

7 $$M_{grad - CAM}^{c} \left[ {l,n} \right] = \sum\limits_{k} {\alpha_{k}^{l,c} F_{k,n}^{l} \left( {AF_{i} ,\tilde{L}_{i} } \right)}$$

with the help of Grad-CAM, we can evaluate how much attention a model pays to each atom during prediction and further visualize the pre-moieties regions of each compound.

## Data Availability

The datasets generated during and/or analyzed during the current study are available in the ChEMBL database [28, 29], https://www.ebi.ac.uk/chembl/.

## Abbreviations

PKFI: Pan-kinase family inhibitor
GCN: Graph convolutional network
Grad-CAM: Gradient-weighted class activation mapping
FDA: Food and Drug Administration
AUROC: Area under the receiver operating characteristic curve
MCC: Matthews correlation coefficient
GAP: Global average pooling
ACC: Accuracy
CNN: Convolutional neural network
